# The Role of the Nucleotides in the Insertion of the bis-Molybdopterin Guanine Dinucleotide Cofactor into apo-Molybdoenzymes

**DOI:** 10.3390/molecules27092993

**Published:** 2022-05-06

**Authors:** Kim Tiedemann, Chantal Iobbi-Nivol, Silke Leimkühler

**Affiliations:** 1 Institute of Biochemistry and Biology, Molecular Enzymology, University of Potsdam, Karl-Liebknecht Str. 24-25, 14476 Potsdam-Golm, Germany; kim.tiedemann@t-online.de; 2 Laboratoire de Bioénergétique et Ingénierie des Protéines, Institut de Microbiologie de la Méditerranée, Centre National de la Recherche Scientifique, Aix-Marseille Université, CEDEX 09, 13402 Marseille, France; iobbi@imm.cnrs.fr

**Keywords:** bis-MGD, chaperone, molybdenum cofactor, TMAO reductase

## Abstract

The role of the GMP nucleotides of the bis-molybdopterin guanine dinucleotide (bis-MGD) cofactor of the DMSO reductase family has long been a subject of discussion. The recent characterization of the bis-molybdopterin (bis-Mo-MPT) cofactor present in the *E. coli* YdhV protein, which differs from bis-MGD solely by the absence of the nucleotides, now enables studying the role of the nucleotides of bis-MGD and bis-MPT cofactors in Moco insertion and the activity of molybdoenzymes in direct comparison. Using the well-known *E. coli* TMAO reductase TorA as a model enzyme for cofactor insertion, we were able to show that the GMP nucleotides of bis-MGD are crucial for the insertion of the bis-MGD cofactor into apo-TorA.

## 1. Introduction

**Figure 1:**
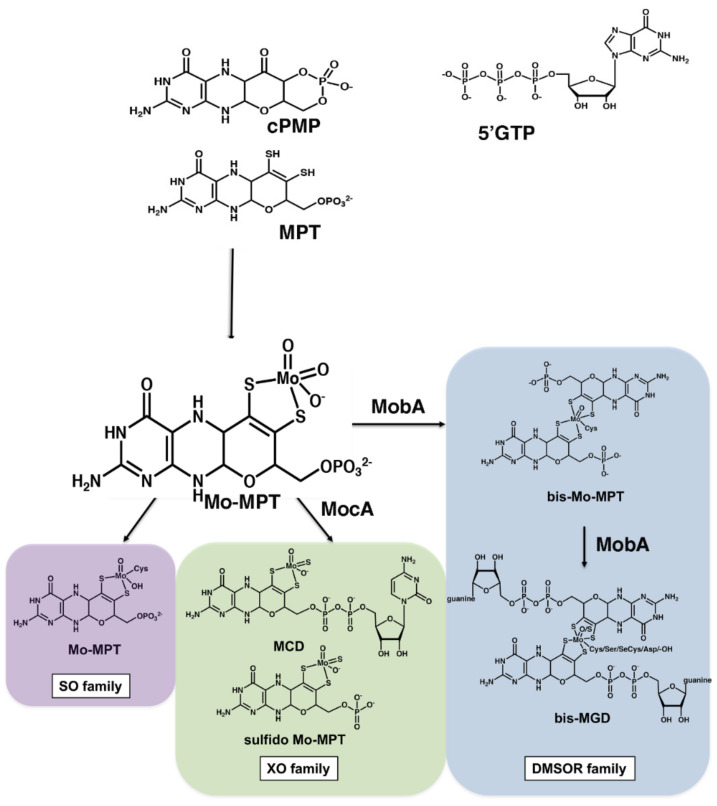
**Different structures of Moco in bacteria.** The basic form of Moco (Mo-MPT) is a 5,6,7,8-tetrahydropyranopterin (MPT) with a dithiolene group coordinating the molybdenum atom in a trioxo form. Moco exists in different variants and is divided into three enzyme families according to the coordination at the molybdenum atom: the SO family, the XO family and the DMSO reductase family. The SO family is characterized by a molybdenum ligation with one oxo, one hydroxide and one cysteine ligand from the protein backbone. In *E. coli*, the XO family contains the sulfurated molybdopterin cytosine dinucleotide cofactor (MCD), while in other bacteria the sulfurated form of Mo-MPT without an additional nucleotide has also been identified. The DMSO reductase family contains two MPTs (bis-Mo-MPT) or two MGDs (bis-MGD) ligated to one molybdenum atom with additional ligands being an O/S and a sixth ligand which can be a serine, a cysteine, a selenocysteine, an aspartate or a hydroxide ligand.

The trace element molybdenum is an essential component for prokaryotes and eukaryotes [1]. It is transported into the cell as molybdate by active high-affinity molybdate transport systems [2]. In biological systems, molybdate is then coordinated to molybdopterin (MPT), a pterin containing a dithiolene group on its 6-alkyl side chain [3], and then forms the molybdenum cofactor (Mo-MPT), or in some bacteria, it is coordinated to the FeS cluster-based iron–molybdenum cofactor (FeMoco) that forms the active site of nitrogenase [4]. With the exception of nitrogenase, the molybdenum cofactor (Moco) is the common element in all molybdoenzymes from different organisms. More than 60 molybdoenzymes binding different forms of Moco have been identified to date [5]. The vast majority of them are found in prokaryotes, while in eukaryotes (plants and animals) only seven are present [6]. The Moco-containing enzymes are categorized on the basis of the different structures of their molybdenum centers, dividing them into three families: the xanthine oxidase (XO) family, the sulfite oxidase (SO) family and the DMSO reductase family (Figure 1) [7]. While eukaryotes produce only enzymes belonging to the sulfite oxidase and xanthine oxidase families, enzymes of all three families are present in prokaryotes, with enzymes of the DMSO reductase family being predominant [8]. The biosynthesis of Moco is highly conserved in all organisms, and it is divided into three general steps according to the stable biosynthetic intermediates that can be isolated [9] (Figure 1). Somewhat surprisingly, not all organisms require the molybdenum cofactor [10,11,12]. The commonly used eukaryotic yeast model organism *Saccharomyces cerevisiae* plays no role in Mo research as *Saccharomyces cerevisiae* contains neither molybdoenzymes nor the components of the Moco biosynthesis pathway. Genome-wide database analyses have revealed a significant number of bacteria and unicellular eukaryotes that do not require molybdenum, while all multicellular eukaryotes are dependent on Mo [12]. In addition, mainly anaerobic archaea and some bacteria are Mo-independent but instead require tungsten (W) for their growth [13]. In the periodic table of elements, W lies directly below Mo and features chemical properties similar to Mo. The biosynthesis of Moco is highly conserved in all organisms, and it is divided into three to four general steps according to the stable biosynthetic intermediates that can be isolated [9] (Figure 1):

1. The synthesis of cyclic pyranopterin monophosphate from 5′GTP (cPMP).
2. Conversion of cPMP into MPT by introduction of two sulfur atoms.
3. Insertion of molybdate to form Moco (Mo-MPT).
4. In most bacteria, Mo-MPT is further modified by covalent attachment of GMP or other nucleotides (like CMP, IMP or AMP) to the terminal phosphate group of MPT via a pyrophosphate link, to form the molybdopterin dinucleotide cofactors [14]. Further, the bis-form containing two MGD or MPT moieties is formed (Figure 1). The roles of these nucleotides in enzyme activity are not clear to date.

After bis-Mo-MPT formation by MobA, for most enzymes in *E. coli*, two GMP moieties from GTP are added to the C4’ phosphates of bis-Mo-MPT, forming the bis-MGD cofactor [15,16]. While MobA was shown to catalyze both reactions, bis-Mo-MPT formation and the addition of the nucleotides to the phosphate groups of both MPTs (Figure 1), the molecular mechanism of bis-Mo-MPT formation and its binding mode to MobA have not been completely resolved to date [17]. The crystal structure of MobA showed that the protein is a monomer containing two domains, in which the N-terminal domain of the molecule adopts a nucleotide (GTP)-binding Rossmann fold and the second domain at the C-terminus harbors a possible MPT binding site [16,18]. Since two Mo-MPT moieties have to be bound to monomeric MobA for bis-Mo-MPT formation, it was suggested that this occurs by using both the MPT and predicted GTP binding sites on one MobA monomer [17]. During this reaction, one molecule of molybdate has to be released when two Mo-MPT molecules are combined. The last steps of Moco modification, including the formation of bis-MGD, prepare the cofactor for insertion into the specific apo-enzymes. The insertion step is catalyzed by Moco-binding molecular chaperones, which bind the respective molybdenum cofactor and insert it into the specific target molybdoenzyme [19]. With a few exceptions, most of the molybdoenzymes have a specific chaperone for Moco insertion [19,20]. One well-studied example is the TorD/TorA system for TMAO reductase in *E. coli*. TorD was shown to be the specific chaperone for TorA [21] and plays a direct role in the insertion of bis-MGD into apo-TorA [22]. During this reaction, TorD interacts with MobA and apo-TorA and acts as a platform, which is required for stabilizing apo-TorA for Moco insertion to avoid a proteolytic attack of the latter. This is consistent with its role as a “facilitator” of the bis-MGD insertion and maturation of the apo-enzyme [21,23,24].

In the *nit-1* assay established by Nason in 1974 [23], the molybdenum cofactor was removed as a low-molecular-weight fraction from denatured Mo-enzymes of mammalian, plant or bacterial origin [23], and after separation, the protein-free Moco fraction was subsequently incorporated into a cofactor-free apo-nitrate reductase from eukaryotes (*nit-1* extract), thereby activating the enzyme. Early studies on Moco from various sources using the *N. crassa nit-1* nitrate reductase reconstitution assay led to the widespread belief in the universality of the cofactor [23]. As more and more purified molybdoenzymes were studied, it was suggested that different forms of Moco exist in some enzymes of prokaryotic origin. For example, the cofactor of *Methanobacterium formicium* formate dehydrogenase was reported to be inactive in the *nit-1* reconstitution assay but was capable of yielding the stable degradation product FormA. In addition, studies by Meyer and coworkers showed that the CO dehydrogenase from *Hydrogenophaga pseudoflava* contained a more complex form of the pterin molecule with an additional nucleotide [24,25]. The study of alkylated pterin derivatives of molybdoenzymes from various sources, in particular studies of the DMSO reductase from *Rhodobacter sphaeroides* [26], gave rise to the identification of a new phosphoric anhydride of molybdopterin and 5′GMP, which was termed molybdopterin guanine dinucleotide cofactor (MGD) [27] (Figure 1). Detailed elucidation of the structure of MGD was based on detailed chemical investigations, showing that the novel pterin contained two phosphates per pterin and that a 5′GMP moiety could be acid hydrolyzed or cleaved from the cofactor by nucleotide pyrophosphatase treatment [28]. The totality of these studies led to the understanding that with the exception of nitrogenase, Moco is the common element in all molybdoenzymes from different organisms. In addition to demonstrating the universality of Moco, the *nit-1* extract assays also demonstrated that Moco is very labile with a lifetime of only a few minutes after release from molybdoenzymes, making chemical characterization of active Moco difficult. Therefore, structural characterization of Moco was achieved through the analysis of the stable degradation products FormA and FormB [29]. The chemical nature of Moco was determined by Rajagopalan and coworkers in 1982, revealing as common component of all Moco structures a reduced pterin with an unusual 6-alkyl side chain consisting of four carbons, a terminal phosphate ester and a unique dithiolene group critical for metal ligation [30]. Later crystal structures of Mo-enzymes have demonstrated the existence of a third pyrano ring between the OH-group at C3’ of the side chain and the pterin C7 atom [31,32,33]. With the closed pyrano ring, a fully reduced and hydrogenated pterin is formed. Because of the unique nature of the pterin in Moco, the metal-free form of the cofactor is called molybdopterin or metal-binding pterin (MPT); the latter reflects the fact that not only Mo but also W can be coordinated by this pterin scaffold producing the Mo-MPT or W-MPT cofactors.

While Nason and coworkers studied the reconstitution of apo-nitrate reductase from *N. crassa*, which belongs to the SO family of molybdoenzymes binding Mo-MPT [23], the first in vitro reconstitution of a bacterial enzyme of the DMSO reductase family with bis-MGD was reported in 1987 for nitrate reductase from *Escherichia coli* and was further optimized in the following decades, showing that the activity of MobA is required for the assembly of the bis-MGD cofactor of nitrate reductase [34].

Previously, we have also established an in vitro system specifically for bis-MGD insertion into apo-TorA. Kaufmann et al. [35], for the first time, tried a reconstitution approach in which bis-MGD was extracted directly from a molybdoenzyme (such as formate dehydrogenase or TMAO reductase) after heat denaturation and apo-TorA was directly incubated with this bis-MGD Moco source without the aid of MobA or TorD [36]. This showed for the first time that bis-MGD was stable and remained intact during the extraction and reinsertion procedure and that MobA and TorD are not essential for bis-MGD insertion in vitro. It was demonstrated in that report that active TorA was obtained after reconstitution, gaining for the first time active TorA after direct reconstitution with extracted bis-MGD. Analysis of the stability of extracted bis-MGD showed that the cofactor was stable for 90 min after extraction under anaerobic conditions, before a reduction in reconstitution efficiency was observed. This showed that the extracted bis-MGD cofactor is more stable under anaerobic conditions than previously anticipated.

Recently, the YdhV protein has been identified as a molybdoenzyme in *E. coli* binding the bis-Mo-MPT cofactor [37]. So far, enzymes containing the bis-MPT cofactor were all described to be tungstoenzymes, harboring tungsten instead of molybdenum at the dithiolenes of MPT [38]. In YdhV, the bis-W-MPT could also be inserted; however, the enzyme was shown to have a preference for molybdenum over tungsten, so YdhV was classified as a molybdoenzyme [37]. The bis-Mo-MPT cofactor of YdhV was revealed to be redox-active, despite the fact that the functional substrate for YdhV still remains to be identified. The failure to identify any substrate for YdhV has been explained by the fact that an inactive enzyme was purified due to the lack of a functional [4Fe-4S] cluster in proximity to the bis-Mo-MPT cofactor. According to a bioinformatic analysis, YdhV has been grouped into an enzyme class containing a so-called “hyper-active” cysteine residue that contributes to [4Fe-4S]-cluster instability during the purification of YdhV even under strict anaerobic conditions [39]. The unexpected presence of a bis-Mo-MPT cofactor in an enzyme of *E. coli* opened an additional route for Moco biosynthesis and expanded the canon of the structurally highly versatile Mocos in molybdoenzymes in *E. coli.*

Since with TorA and YdhV we have now two systems in hand which can be used for the reconstitution with different forms of the molybdenum cofactor, we wanted to investigate the role of the nucleotides in the insertion and activity of these molybdoenzymes. Several structural features of the bis-MGD cofactor of the DMSO reductase family have been analyzed in the last years, including the role of the Mo ligands and even the role of the amino acids in the first coordination sphere of the central Mo ion. Recently, Rothery et al. observed a correlation between the geometry of the pterin and the oxidation state of the enzyme [40]. Later, Stolz et al. investigated how the amino acid environment of the pterins affects the enzymatic activity of Nar nitrate reductase of *E. coli*, confirming the involvement of the pterins in the electrochemical properties of the central Mo^VI/IV^ [41]. Investigations that have been missing are studies on the role of the nucleotides on the pterin ligand in Moco insertion and the activity of molybdoenzymes.

To study the role of the nucleotides of the bis-MGD cofactor, we decided to use a model enzyme in form of the *E. coli* TMAO reductase TorA, which contains bis-MGD as the sole prosthetic group [21,42]. Previously, it had been shown that apo-TorA can be reconstituted with the bis-MGD cofactor either isolated from enzymes of the same enzyme family [36] or synthesized in vitro using proteins from the Moco biosynthesis machinery [17,43]. In this study, the binding of the bis-MGD cofactor to apo-TorA is compared to the binding of the recently identified bis-Mo-MPT cofactor from *E. coli* YdhV, which has the same central structure as bis-MGD but lacks the GMP nucleotides (Figure 1) [37]. In addition to apo-TorA, two other proteins were selected as Moco acceptors in this study: apo-YdhV, which binds the bis-MPT cofactor, and BSA, which does not contain a Moco-binding pocket and therefore is used as a negative control to test for unspecific Moco binding.

## 2. Results

### 2.1. The Specificity of the Cofactor Insertion into the Respective Target Enzyme: Production of bis-MGD and bis-Mo-MPT

**Scheme 1:**
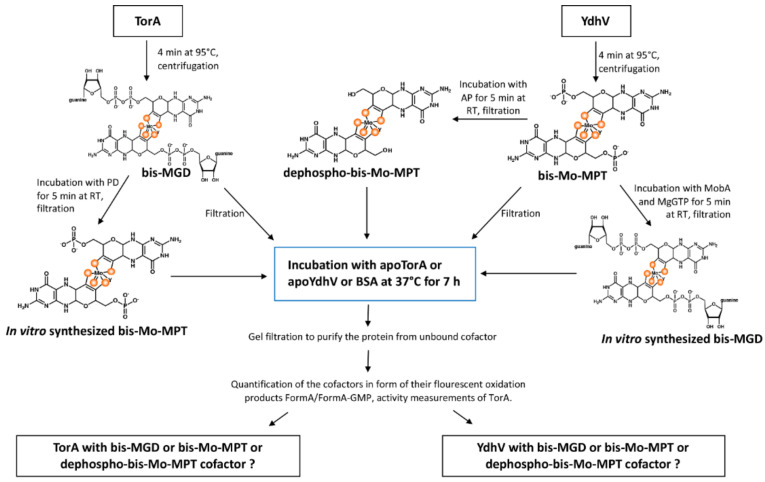
Overview of the different incubations for insertion of the different cofactor forms into apo-TorA or Apo-VdhV. Details are given in the text and Section 3.

To analyze whether bis-MGD and bis-Mo-MPT are functionally identical and result in enzymes with comparable activities after insertion into an apo-molybdoenzyme, we extracted bis-MGD from TorA and bis-Mo-MPT from YdhV by heat treatment. Extracted cofactors were then inserted into both apo-TorA and apo-YdhV by using the previously established in vitro reconstitution system (Scheme 1) [35]. All steps of Moco extraction and modifications and apoenzyme reconstitution were carried out under anoxic conditions using purified apo-proteins as described in Section 3. To ensure that the bis-Mo-MPT cofactor was extracted from YdhV in a functionally competent form, we reinserted the extracted cofactor into apo-YdhV and tested it for insertion, since no activity test is available for YdhV.

**Figure 2:**
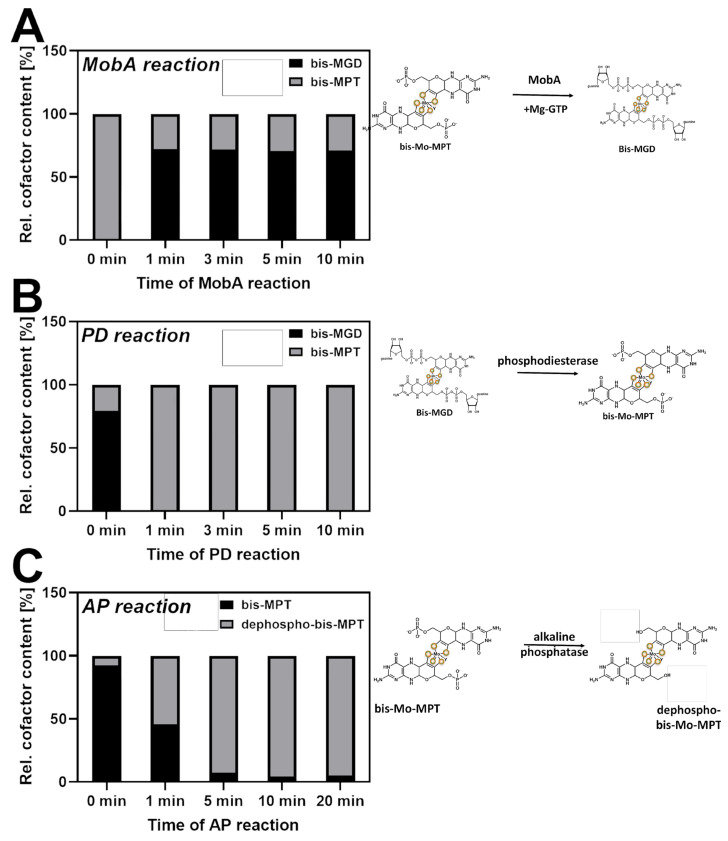
Conversion efficiency over time for enzymatic conversion of cofactors. (**A**) Conversion of bis-MPT from 30 µM YdhV to bis-MGD catalyzed by 3 µM MobA in presence of 1 mM GTP and 1 mM MgCl_2_. (**B**) Conversion of bis-MGD from 30 µM wtTorA to bis-MPT catalyzed by 10 U/mL phosphodiesterase I (PD). (**C**) Conversion of bis-MPT from 30 µM YdhV to dephospho-bis-MPT catalyzed by 10 U/mL alkaline phosphatase (AP). The reactions were terminated by the addition of acidic I_2_/KI solution. The cofactors were quantified in form of their fluorescent oxidation products FormA-GMP and dephospho-FormA. On the right-hand side next to the panels, a scheme is shown of the enzymatic reaction that was performed to convert the cofactor into a different form.

Further, we converted bis-Mo-MPT into bis-MGD by the attachment of nucleotides using MobA and Mg-GTP, and we converted bis-MGD to bis-Mo-MPT by cleavage of the nucleotides with phosphodiesterase I (PD) (Figure 2A,B). The conversion rate of bis-Mo-MPT to bis-MGD was 66% (Figure 2A) while 99% of bis-MGD was converted into bis-Mo-MPT (Figure 2B).

In addition, we used BSA as background control for unspecific Moco binding.

**Figure 3:**
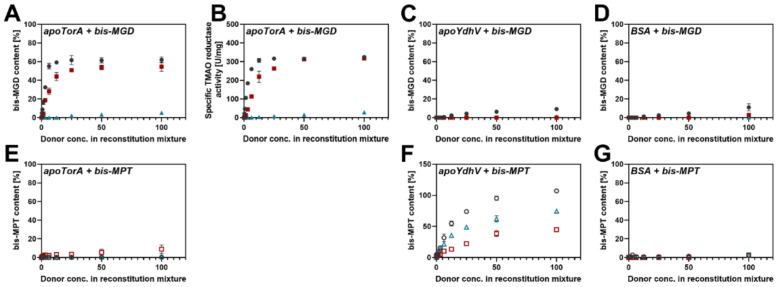
Cofactor binding to apo-TorA, apo-YdhV and BSA. The bis-MGD content of 1.3 µM apo-TorA (**A**), apo-YdhV (**C**) and BSA (**D**) is shown after incubation with bis-MGD from different sources: bis-MGD isolated from wtTorA as donor (circles), bis-MGD formed in vitro out of bis-MPT in a MobA-catalyzed reaction (squares), residual bis-MGD after conversion into bis-MPT in a PD-catalyzed reaction (triangles). Bis-MGD was detected in form of its fluorescent oxidation product FormA-GMP. Panel (**B**) represents the specific TMAO reductase activity of the samples in panel A. The bis-Mo-MPT content of 1.3 µM apo-TorA (**E**), apo-YdhV (**F**) and BSA (**G**) is shown after incubation with bis-Mo-MPT from different sources: bis-Mo-MPT isolated from YdhV as donor (circles), residual bis-Mo-MPT after conversion into bis-MGD in a MobA-catalyzed reaction (squares), bis-Mo-MPT formed in vitro out of bis-MGD in a PD-catalyzed reaction (triangles). Bis-Mo-MPT was detected in form of its fluorescent oxidation product dephospho-FormA. No TMAO reductase activity was detected for these samples.

**Bis-MGD insertion into apo-TorA, apo-YdhV and BSA:** The results of the described bis-MGD insertion are depicted in Figure 3. Bis-MGD extracted from wtTorA was readily inserted into apo-TorA (Figure 3A). Between 0 and 6.25 µM of donor, the bis-MGD binding to apo-TorA increased in a hyperbolic saturation curve, and at 25 µM of donor, the bis-MGD content reached saturation. The maximum bis-MGD saturation of 61.9% shows that 38% of apo-TorA was not in a competent form for cofactor insertion, which is consistent with previous results on other molybdoenzymes (Figure 2A) [43,44].

When bis-MGD was formed in vitro in a MobA-catalyzed reaction by the addition of GMP nucleotides to extracted bis-Mo-MPT (Figure 2A), the cofactor was as readily inserted as a direct bis-MGD source (Figure 3A). In this case, a similar saturation curve for bis-MGD insertion was obtained, but higher donor concentrations were required for the same cofactor loading since less cofactor was available due to the partially incomplete cofactor conversion of 66%.

Finally, when wtTorA-extracted bis-MGD was subjected to PD hydrolysis, almost no bis-MGD insertion into apo-TorA was observed, showing that the bis-MGD cofactor was successfully converted into bis-Mo-MPT and was therefore unavailable for incorporation into apo-TorA (Figure 3A).

The specific TMAO reductase activities were measured for all apo-TorA reconstitution samples (Figure 3B). The bis-MGD content and the TMAO reductase activity were found to correlate well, which shows that the cofactor is both intact and correctly inserted into apo-TorA (Figure 3A,B). No differences in activities were observed between the bis-MGD cofactor extracted from wtTorA and the bis-MGD cofactor synthesized from bis-Mo-MPT (Figure 3B).

Furthermore, the binding of bis-MGD to apo-YdhV and BSA was investigated. Unaffected by the origin of the bis-MGD cofactor (being directly isolated from wtTorA or obtained through MobA catalysis), only a neglectable amount of bis-MGD was bound to apo-YdhV and BSA (Figure 3C,D). This led to the conclusion that the bis-MGD cofactor does not bind unspecifically to a protein such as BSA, or binds unspecifically only to a minor extent, and is not inserted into apo-YdhV.

### 2.2. bis-Mo-MPT Insertion into apo-TorA, apo-YdhV and BSA

Similar reconstitution experiments were performed except that bis-Mo-MPT from either wtYdhV extraction or bis-MGD conversion by PD was used as a cofactor source. The results shown in Figure 3F reveal that according to the origin of the bis-Mo-MPT source, the reconstitution level of apo-YdhV is different. It is optimal when it was directly extracted from wtYdhV with almost all the apo-enzyme matured at 50 µM of donor. During MobA-dependent conversion of bis-Mo-MPT into bis-MGD, 34% of bis-Mo-MPT is not converted (Figure 2A). When this residual bis-Mo-MPT is used in the in vitro YdhV maturation assay, at low donor concentration (to 6.25 µM) the maturation rate is similar to that of bis-Mo-MPT directly extracted, but the plateau reaches only 45% of reconstituted YdhV. This indicates probably that the bis-Mo-MPT source is limiting in this case. In contrast, when bis-Mo-MPT was produced in vitro from bis-MGD in a PD-catalyzed reaction, at low and high donor concentrations, maturation of the apo-YdhV is less efficient than with bis-Mo-MPT directly extracted and the plateau reaches only 75% of matured enzyme. We observed during our experiments that bis-Mo-MPT is less stable than bis-MGD, and even though the conversion reaction rate of the phosphodiesterase reaction was 99%, we probably lost part of the bis-Mo-MPT during the incubation reaction.

When similar experiments were performed with apo-TorA, whatever the bis-Mo-MPT source, no enzyme maturation occurred, indicating that bis-Mo-MPT cannot be inserted into apo-TorA. The same results were obtained when BSA was used to reveal unspecific cofactor binding (Figure 3E,G for apo-TorA and BSA, respectively).

### 2.3. Attempts to Facilitate bis-MPT Insertion into apo-TorA

**Figure 4:**
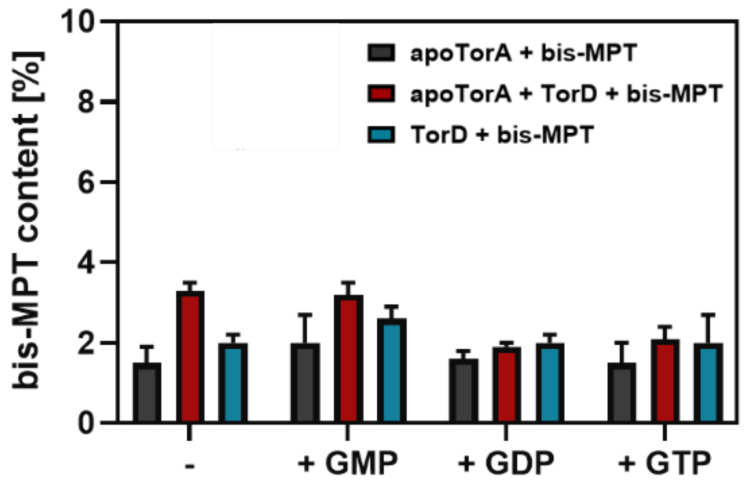
Effect of the presence of TorD and effect of free nucleotides in the reconstitution mixture on the insertion of bis-Mo-MPT into apo-TorA. Here, 1.3 µM apo-TorA, 2 µM TorD or both were incubated with bis-Mo-MPT isolated from 30 µM YdhV, and 50 mM GMP, GDP or GTP was added prior to the reconstitution mixture. Bis-Mo-MPT was detected in form of its fluorescent oxidation product dephospho-FormA.

As bis-Mo-MPT itself was not binding to apo-TorA, we thought of missing parts for bis-Mo-MPT insertion into apo-TorA. Since the nucleotides seem to be crucial for the insertion step, we tested whether nucleotides prebound to apo-TorA would help bis-Mo-MPT insertion [43]. Therefore, 50 mM GMP was added prior to the reconstitution mixture in an attempt to fill the nucleotide-binding pocket and stabilize the protein. Additionally, 50 mM GDP or GTP was used as a negative control since bis-MPT would not fit into the pocket with GDP or GTP bound to the apo-protein. Furthermore, 2 µM of the private chaperone of TorA, TorD, was added. TorD has been shown to both stabilize apo-TorA and aid the cofactor insertion via an interaction with the core of the molybdoprotein [20,21]. However, as depicted in Figure 4, neither TorD nor GMP, GDP or GTP led to a significant increase in bis-Mo-MPT incorporation into apo-TorA. Since a Moco-binding chaperone for YdhV has not been identified so far, the reconstitution assays for YdhV were performed without the addition of a Moco-binding chaperone.

### 2.4. Production of Dephospho-bis-Mo-MPT and Insertion into apo-TorA and apo-YdhV

The bis-Mo-MPT cofactor from YdhV was not inserted into apo-TorA despite the overall similarity of the shared structural features of the bis-Mo-MPT core. This raised the question of whether the negative charges of the two terminal phosphate groups of the bis-Mo-MPT cofactor might hinder its insertion into the apo-TorA. Therefore, we cleaved the terminal phosphate groups of bis-Mo-MPT by treatment with alkaline phosphatase. To investigate the binding of dephospho-bis-Mo-MPT, bis-Mo-MPT extracted from YdhV was incubated with alkaline phosphatase (AP) for 5 min at RT to catalyze the hydrolysis of the phosphate group. The conversion rate of this reaction was 95% (Figure 2C).

**Figure 5:**
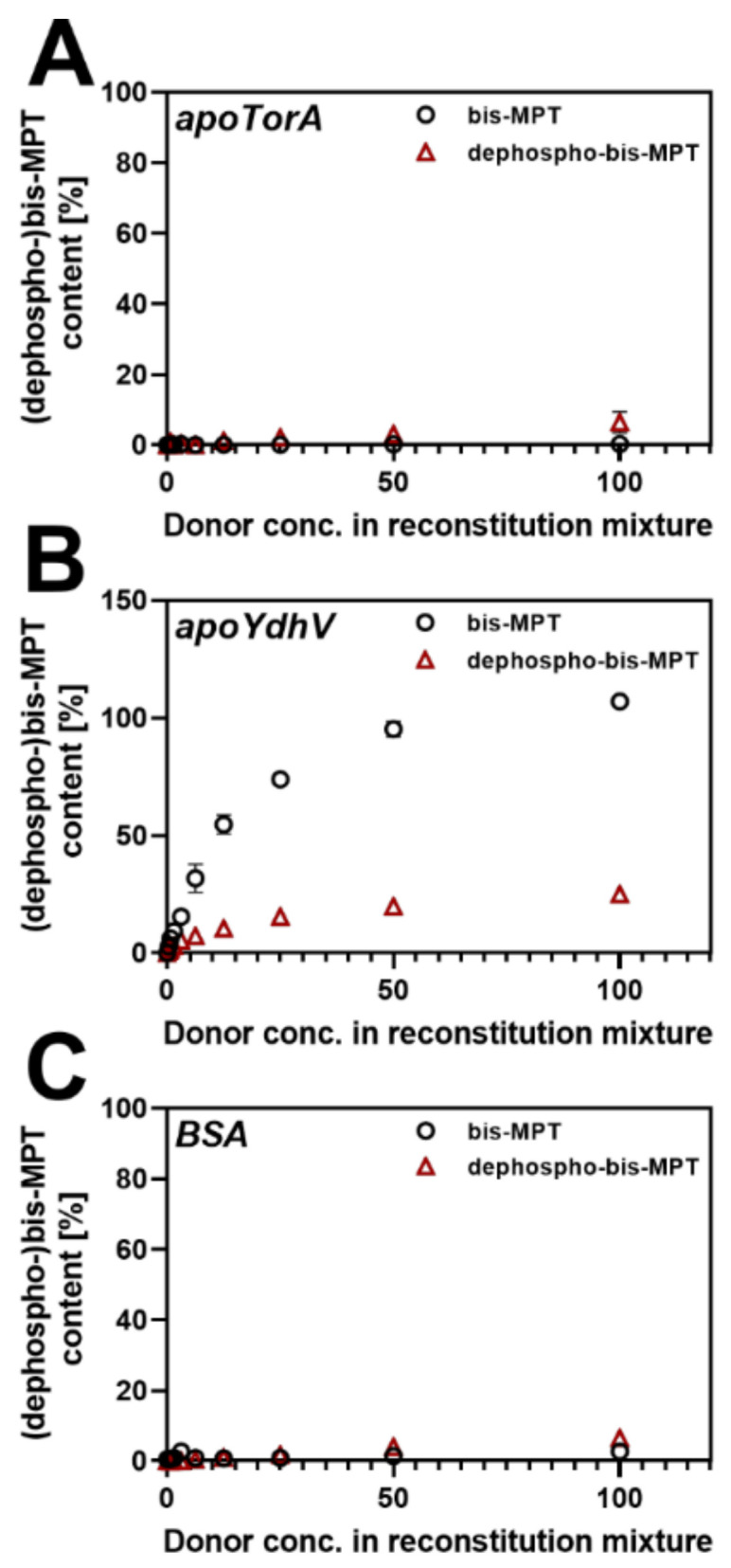
Bis-Mo-MPT content of 1.3 µM apo-TorA (**A**), apo-YdhV (**B**) and BSA (**C**) incubated with bis-Mo-MPT or dephospho-bis-MPT. Bis-Mo-MPT (circles) was directly isolated from YdhV. Dephospho-bis-Mo-MPT (triangles) was obtained after in vitro hydrolysis of the terminal bis-Mo-MPT phosphate group by alkaline phosphatase (AP). Bis-MPT and dephospho-bis-MPT were detected in form of their fluorescent oxidation product dephospho-FormA.

As depicted in Figure 5, dephospho-bis-Mo-MPT showed no improved insertion into apo-TorA in comparison to bis-Mo-MPT. Apo-TorA contained 6.5% dephospho-bis-MPT at 100 µM of donor concentration (Figure 5A), but this binding seemed to be unspecific since the same amount of the truncated cofactor was bound to BSA (Figure 5C). Nevertheless, we tested if dephospho-bis-MPT would bind at higher concentrations. Thus, apo-TorA was incubated with 250 µM of dephospho-bis-MPT, but no improvement of dephospho-bis-MPT insertion into apo-TorA was observed. Therefore, we conclude that while the nucleotide is crucial for bis-MGD insertion, the phosphate group does not hinder the insertion of bis-Mo-MPT into apo-TorA. Furthermore, the binding of the dephospho-bis-Mo-MT cofactor to apo-YdhV was heavily impaired when the phosphate group was cleaved (Figure 5B). At 100 µM of donor, only 25.1% of dephospho-bis-MPT was bound to apo-YdhV, which is only a fourth compared to intact bis-Mo-MPT (Figure 5B). This finding suggests that the phosphate group is crucial for the binding of bis-Mo-MPT to apo-YdhV.

## 3. Materials and Methods

### 3.1. Expression and Purification Conditions

Apo-TorA, wtTorA (both from pJF119EH [21]), TorD (from pET28TorD [21]) and MobA (from pCT800A [43]) were expressed and purified as described before [36]. Apo-YdhV (from SR153 [37]) was expressed in *E. coli* RK5200 with 20 µM isopropyl-β-D-thiogalactopyranoside (IPTG) and 150 µg/mL ampicillin. After inoculation with an aerobically grown preculture (16 h, 37 °C, 200 rpm) at a 1:500 dilution, cells were grown for 24 h at 30 °C and 130 rpm under aerobic conditions. Wild-type YdhV (wtYdhV) was expressed in *E. coli* TP1000 with 135 µM IPTG, 150 µM ampicillin and 100 mM Na_2_MoO_4_. After inoculation with aerobically grown preculture at a 1:50 dilution, cells were grown anaerobically in closed flasks for 24 h at 30 °C.

### 3.2. Protein Purification

After harvesting, cells were resuspended in 50 mM NaH_2_PO_4_ buffer containing 10 mM of imidazole. For purification under aerobic conditions, cell lysis was performed on a cell disruptor system (Constant Systems LTD, Northants, UK). The cleared lysate was loaded onto a self-packed Ni-nitrilotriacetate (Ni-NTA) column with 0.5–0.7 mL matrix per liter of cell culture. After washing with 10 column volumes of buffer containing 10 mM imidazole and 20 column volumes of buffer containing 20 mM imidazole, the protein was eluted with 250 mM imidazole buffer. The protein buffer was exchanged to 100 mM Tris-HCl pH 7.2 using PD-10 columns (GE Healthcare, Little Chalfont, UK). Apo-TorA, TorD, MobA and apo-YdhV were further purified via size exclusion chromatography on a 16/600 Superdex 200 prep grade column (GE Healthcare, Little Chalfont, UK). After concentrating the samples, proteins were aliquoted in small fractions, frozen in liquid nitrogen and stored at −80 °C. WtTorA and wtYdhV were both purified under anaerobic conditions in a glovebox (Coy Laboratory Products, Grass Lake, MI, USA). Cell lysis was performed via ultrasound sonification. All other purification steps were performed as described before for aerobic purification, except less Ni-NTA resin was used (0.2–0.3 mL resin per liter of cell culture).

### 3.3. Cofactor Analysis

Quantification of the metal content was performed using a PerkinElmer (Waltham, MA, USA) LifeSciences Optima 2100DV inductive coupled plasma optical emission spectrometer (ICP-OES) following the established procedure [45]. The bis-MGD cofactor and the bis-Mo-MPT cofactor content were determined by HPLC after their conversion into the stable oxidized fluorescent degradation products FormA-GMP and FormA as described previously [35]. In this method, 25 µL acidic KI/I_2_ solution was incubated with 200 µL sample overnight. After centrifugation, 27.5 µL 1% ascorbic acid and 100 µL 1 M Tris were added to the supernatant, which was subsequently incubated with 1 U AP for 30 min. After the addition of 10 µL of 50% acetic acid, the samples were applied to a C18 reversed-phase HPLC and fluorescence was monitored using an Agilent 1100 series fluorescence detector (Agilent Technologies, Santa Clara, CA, USA) with 383 nm excitation and 450 nm emission wavelengths. As a standard for FormA-GMP, the FormA-GMP signal of fully cofactor-saturated wtTorA was set to 100%. As a standard for FormA, FormA-GMP from wtTorA was incubated at 95 °C for 40 min to convert FormA-GMP into FormA, which was set to 100%.

### 3.4. Activity Assay

The enzymatic activity of TorA was measured under anaerobic conditions by monitoring the oxidation of pre-reduced benzyl viologen at 600 nm. The assay was conducted in 100 mM Sörensen phosphate buffer pH 6.5 containing 7.5 mM TMAO and 0.4 mM BV, which was reduced by the addition of sodium dithionite. Measurement was started after the addition of the respective enzyme sample.

### 3.5. Reconstitution Assay

In vitro reconstitution of apo-TorA was performed in 100 mM Tris-HCl buffer pH 7.2 under anaerobic conditions in a glove box. The respective Moco-containing donor protein (wtTorA or wtYdhV) was incubated at 95 °C for 4 min followed by a centrifugation step. The supernatant was filtered through 10 kDa Amicon filters and incubated with 1.3 µM apo-TorA for 7 h at 37 °C. The reconstitution/binding assays for apo-YdhV and BSA were carried out following the same protocol. In some cases, the originally isolated cofactor was modified prior to incubation with apo-TorA, apo-YdhV or BSA. To convert bis-Mo-MPT into bis-MGD, 3 µM MobA, 1 mM 5′GTP and 1 mM MgCl_2_ were incubated with the bis-Mo-MPT-containing supernatant for 5 min at RT followed by a short heating period and filtration of the solution. For conversion of bis-MGD to bis-Mo-MPT, 10 U/mL phosphodiesterase I (PD, Type IV from Crotalus atrox, Sigma-Aldrich chemie GmbH, 82024 Taufkirchen, Germany) was incubated with the bis-MGD-containing supernatant for 5 min for hydrolysis of GMP followed by filtration of the solution. For the truncation of the terminal phosphate groups of bis-Mo-MPT, the supernatant was incubated for 5 min at RT with 10 U/mL alkaline phosphatase (AP, Thermo Scientific company) followed by a filtration step.

To verify the cofactor conversion, HPLC-based cofactor analysis (described above) was used to provide information about the cofactor type and content in the supernatant. To verify the AP-catalyzed conversion of bis-Mo-MPT to dephospho-bis-Mo-MPT, no additional AP was added in the cofactor analysis method.

After incubation with the respective cofactor, apo-TorA, apo-YdhV and BSA were purified from low-molecular-weight compounds and excess cofactor via gel filtration on a Nick Sephadex column (GE Healthcare, Little Chalfont, UK) in 100 mM Tris-HCl pH 7.2 buffer before quantification of the cofactor content.

## 4. Discussion

One of the last remaining questions in Moco biosynthesis is why some enzymes bind the dinucleotide variant of the cofactor while others do not. This question has come more into focus with the identification of the presence of the bis-Mo-MPT cofactor present in YdhV in *E. coli* [37]. One hypothesis in the past was that the attachment of nucleotides provides a level of regulation by the availability of the *mobAB* gene products for nucleotide insertion [46]. However, an investigation of the regulation of the *mobAB* locus in *R. capsulatus* or *E. coli* did not reveal any insights into a regulatory effect of bis-MGD biosynthesis for cofactor availability [42,46]. Another suggestion was thus a stabilizing role of the nucleotides for the bis-MGD-free apo-enzyme of *R. capsulatus* [43]. However, even with the guanine moieties present, the apo-enzyme was less stable than the holoenzyme. In this paper, we focused on the role of the terminal GMP nucleotides in the bis-MGD cofactor in insertion into apo-enzymes of the DMSO reductase family, choosing apo-TorA as a model enzyme. To compare bis-MGD to a cofactor, which is structurally similar but lacks the nucleotides, bis-Mo-MPT from YdhV was used. Apo-TorA was found to incorporate the bis-MGD cofactor either provided from TorA or produced in vitro from bis-Mo-MPT in a MobA-dependent manner. The specific TMAO reductase activity matched the amount of bound bis-MGD, which confirmed that the cofactor was intact and correctly inserted. We also observed that the bis-Mo-MPT cofactor was not inserted into apo-TorA even when it was formed from bis-MGD via cleavage of GMP with phosphodiesterase. As a potential reduced stability of the bis-Mo-MPT cofactor in vitro might have led to the inability of apo-TorA incorporation, we inserted the cofactor into apo-YdhV to confirm its structural integrity and stability. As expected, apo-YdhV did not bind the bigger bis-MGD cofactor in its binding pocket tailored for bis-Mo-MPT. BSA, which does not contain any Moco-binding pocket, was selected as a negative control for cofactor binding and did not bind to bis-MPT or bis-MGD. Therefore, the unspecific binding of the cofactors was neglectable in our experimental setup. Here, it was also revealed that the phosphate group is a crucial component for the insertion of the bis-Mo-MPT cofactor into apo-YdhV. In 2000, Temple and Rajagopalan suggested a stabilizing role of the nucleotides for the bis-MGD-free apo-enzyme [43]. They were able to purify a GTP-containing DMSO reductase purified from an *E. coli* ∆*mobAB* mutant strain. However, in their study, it remained unclear whether the guanine moieties present in ∆*mobA*B DMSOR play any role in cofactor biosynthesis and insertion in vivo. However, the absence of MPT in apo-DMSOR showed that occupancy of the guanine nucleotide-binding site does not by itself create the MPT binding site, since no MPT was found to be bound to the protein. They showed that the guanine moieties incorporated into the activated DMSOR originated from the GTP added to the in vitro reconstitution mixture containing MobA and GTP, as demonstrated by the use of radiolabeled GTP in the assay. Although the GMP and GDP are bound to apo-DMSOR strongly enough to be present stoichiometrically in the purified protein, the data presented in their study showed that they can be replaced in the process of inserting the in vitro assembled bis-Mo-MGD cofactor into the protein. It may be that the guanine moieties found in ∆*mobAB* and ∆*mobA* DMSOR served to stabilize the protein before cofactor insertion, and one proposed role for the potential DMSOR chaperone DorD would be to catalyze the rapid exchange of the bound nucleotides for the bis-MGD molybdenum cofactor. It also remained possible from their study that, in vivo, the apo-protein might interact with its specific chaperone to prevent intermediate binding of GMP and GDP. This was not tested in their study, since the chaperone DorD was not coexpressed. Even with the guanine moieties present, heat sensitivity and overall low yield indicated that ∆*mobAB* DMSOR is less stable than the holoenzyme. However, the role of the chaperone might not be completely crucial for the insertion of the cofactor, since in our reconstitution assays, the cofactors were also inserted without the help of the chaperone.

In addition, other enzymes were found to bind nucleotides in the absence of MPT. Hänzelmann et al. (1998) [47] purified CO dehydrogenase, a molybdopterin cytosine dinucleotide (MCD)-containing enzyme, from *Hydrogenophaga pseudoflava* grown in the absence of molybdenum or in the presence of tungstate, and they found that it was devoid of MCD while still containing a stoichiometric amount of cytidine moieties, including CDP, dCDP, CMP, dCMP, CTP and dCTP (listed in order of abundance). Since only GMP and GDP were found in ∆*mobAB* DMSOR, this may indicate a greater specificity over CTP nucleotides in the guanosine-binding pocket of DMSOR [43]. The presence or absence of guanine moieties in *E. coli* DMSOR and NR expressed in the absence of MobA was not investigated in the studies mentioned earlier [48,49,50]. In our study, however, no nucleotides were bound to apo-TorA or were shown to facilitate the insertion of the bis-Mo-MPT cofactor into the apoenzyme. The influence on the stability, therefore, was not investigated in our study, since the nucleotide-containing protein was not available.

In conclusion, GMP is crucial for the binding of the bis-MGD cofactor to apo-TorA. What might be the reason for that? Czjzek et al. solved the crystal structure of *Shewanella massilia* TorA in 1998 [51] and reported the possible hydrogen bond interactions of the protein with the bis-MGD cofactor. In numbers, 21 possible hydrogen bonds are formed between the protein and the terminal GMP group, and 28 possible hydrogen bonds are formed between the protein and the pterin rings and the molybdenum center including its ligands. It might be speculated that more than half of the hydrogen bonds should still be enough as a binding pocket to insert a cofactor as complex as bis-Mo-MPT. Since it waits to incorporate a rather large cofactor, apo-TorA is widely believed to be in an “open” conformational state [20], in which the enzymatic binding pocket is stretched and opened towards the outside. In this state, the edges of the binding pocket, where GMP would bind, are presumably already defined while the center part is not. The binding of the terminal GMP groups might act as the anchor for the insertion of the bis-MPT part of the cofactor and lead to a conformational change, opening the binding pocket for the pterin parts of the cofactor enabling the rest of the cofactor to form hydrogen bonds with the protein. Therefore, in our model, the binding of the terminal GMP groups is the first step in the bis-MGD incorporation into enzymes of the DMSO reductase family and thus is crucial for the binding of the whole cofactor anchor. Specific chaperones also have a role in the insertion of the bis-MGD into apoprotein. For TorA, it was established that its specific chaperone TorD interacts with two distinct regions of the Moco-free apo form of TorA, which encompasses the signal sequence at the N-terminal part of TorA and a binding site in the core of the TorA apoprotein [19]. By binding to the core of the apoprotein, TorD induces a conformational change of apo-TorA that becomes consequently competent for bis-Mo-MPT moiety insertion [52]. We propose, by this model, that the nucleotides are inserted first by TorD into apo-TorA, which then induces a conformational change that facilitates the binding of the bis-MPT moiety; the charges of the phosphates are also likely crucial for the tight interaction of the bis-MGD in the binding pocket of TorA.

Analogously to this, the binding of the bis-Mo-MPT phosphate groups is the first step of incorporation of bis-MPT into apo-YdhV and is important for the binding of the cofactor, but not essential since dephospho-bis-Mo-MPT was still inserted, although with reduced efficiency.

## Data Availability

Not applicable.

## Sample Availability

Samples of the plasmids used available from the authors.
